# Mitotic-Spindle Organizing Protein MztA Mediates Septation Signaling by Suppressing the Regulatory Subunit of Protein Phosphatase 2A-ParA in *Aspergillus nidulans*

**DOI:** 10.3389/fmicb.2018.00988

**Published:** 2018-05-08

**Authors:** Ping Jiang, Shujun Zheng, Ling Lu

**Affiliations:** Jiangsu Key Laboratory for Microbes and Functional Genomics, Jiangsu Engineering and Technology Research Center for Microbiology, College of Life Sciences, Nanjing Normal University, Nanjing, China

**Keywords:** ParA, MztA, PP2A, suppressor, septation, *Aspergillus nidulans*

## Abstract

The proper timing and positioning of cytokinesis/septation is crucial for hyphal growth and conidiation in *Aspergillus nidulans*. The septation initiation network (SIN) components are a conserved spindle pole body (SPB) localized signaling cascade and the terminal kinase complex SidB-MobA, which must localize on the SPB in this pathway to trigger septation/cytokinesis. The regulatory subunit of phosphatase PP2A-ParA has been identified to be a negative regulator capable of inactivating the SIN. However, little is known about how ParA regulates the SIN pathway and whether ParA regulates the septum formation process through affecting the SPB-localized SIN proteins. In this study, through RNA-Seq and genetic approaches, we identified a new positive septation regulator, a putative mitotic-spindle organizing protein and a yeast Mzt1 homolog MztA, which acts antagonistically toward PP2A-ParA to coordinately regulate the SPB-localized SIN proteins SidB-MobA during septation. These findings imply that regulators, phosphatase PP2A-ParA and MztA counteract the septation function probably through balancing the polymerization and depolymerization of microtubules at the SPB.

## Introduction

In all eukaryotic organisms, cytokinesis is the cell division process after the nuclear division of mitosis in which the cytoplasm of a cell is physically partitioned into two. Although organisms of different kingdoms have developed unique mechanisms to execute cytokinesis, signals that trigger the onset of cytokinesis are evolutionarily conserved (Wolkow et al., 1996; Krapp and Simanis, 2008; Seiler and Justa-Schuch, 2010). Many lines of evidence have identified that the conserved mitotic exit network (MEN) components, which are tightly connected with cytokinesis, exist in the budding yeast *Saccharomyces cerevisiae* and mammalian systems (Foltman and Sanchez-Diaz, 2017; Guo and Segal, 2017; Renicke et al., 2017; Scarfone and Piatti, 2017). A pathway homologous to the MEN, termed the septation initiation network (SIN), has also been found to couple mitotic exit with cytokinesis in the fission yeast, *Schizosaccharomyces pombe* and the model filamentous fungus *A. nidulans* (Barr and Gruneberg, 2007; Csikasz-Nagy et al., 2007; Zhong et al., 2012; Simanis, 2015). Genetic analysis has identified that the SIN components are a conserved spindle pole body (SPB) localized signaling cascade, which uses SPB, the functional counterpart of the centrosome in yeasts as a scaffold from which to initiate signaling (Magidson et al., 2006; Lengefeld et al., 2017; Rüthnick et al., 2017). The core SIN network is composed of a GTPase, three protein kinases, an inhibitory GAP complex, and a scaffold complex that anchors the pathway to SPBs (Johnson et al., 2012). Among them, Sid2-Mob1 is the terminal kinase complex in the pathway, which transitions from SPB to the cell division site in anaphase to drive cytokinesis. During the completion of mitosis, SidB and MobA appear at both the SPB and the division site. As the transfer factors, SidB and MobA have the capacity to form a ring to complete septation initiation at the SPB (Harris et al., 1994; Sparks et al., 1999; Kim et al., 2006; Eslami et al., 2014). Therefore, SPB-localized SIN proteins especially Sid2-Mob1 in *S. pombe* and SidB-MobA in *A. nidulans* must transmit signals through the cascade to trigger cytokinesis so that the SPB works as a signaling hub for this process.

In addition, the SPB also serves as a microtubule-organizing center (MTOC), to seed the polymerization of the mitotic spindle and to provide the nucleating machinery for regulating microtubule attachment and dynamics and establishing microtubule polarity (Zekert et al., 2010; Takeshita and Fischer, 2011; Kilmartin, 2014; Wieczorek et al., 2015). The major regulator of microtubules (MTs) nucleation is the γ-tubulin ring complex (γ-TuRC), which caps the minus ends of the MTs and facilitates directional MTs nucleation. Thus, MTs are dynamic polymers transiting between polymerization and depolymerization (Xiong and Oakley, 2009; Suri et al., 2014; Walia et al., 2014). Moreover, signals transmitting SPB-localized SIN proteins through the cascade to trigger cytokinesis need spatial and temporal control of MT-dependent cellular restructuring events (Robinson and Spudich, 2004; Rankin and Wordeman, 2010; Zhou et al., 2010; Ngo et al., 2016). In human tissue, a protein called the mitotic-spindle organizing protein (MOZART1) that interacts with γ-TuRC has been identified to promote the polymerization of the microtubule and then give rise to branched microtubules (Hutchins et al., 2010; Teixidó-Travesa et al., 2010; Cukier et al., 2017). However, the knowledge of whether the major regulator γ -TuRC of MTs nucleation affects cytokinesis is still limited.

SIN signaling requires three protein kinases for its function and thus the phosphorylation/dephosphorylation reactions play important roles by regulating protein activity and subcellular localization in the septum formation processes (Bruno et al., 2001; Sharpless and Harris, 2002; Ramsubramaniam et al., 2014; Rusin et al., 2017). Three main classical protein phosphatases including serine/threonine phosphatases, protein tyrosine phosphatases and the aspartate-based catalysis protein phosphatase coordinate and regulate the septation signaling pathway through counteracting several protein kinases (Son and Osmani, 2009). Phosphatase PP2A is a major intracellular protein phosphatase, which contains three subunits, namely, a catalytic subunit, a structural subunit and several regulatory subunits. Previous studies indicate that the regulatory subunits PP2A-Par1 and PP2A-Pab1 in yeasts are negative regulators that inactivate the SIN (Jiang and Hallberg, 2001; Lahoz et al., 2010; Goyal and Simanis, 2012). However, unlike in yeasts, the regulatory subunit of PP2A-ParA counteracts PabA during the septation process. In addition, ParA localizes to the septum site and the deletion of *parA* causes hyperseptation, while the overexpression of *parA* abolishes septum formation (Zhong et al., 2014). This suggests that ParA functions as a negative factor in regulating the SIN pathway. However, little is known about how ParA regulates the SIN pathway and whether ParA regulates septum formation process by affecting the SPB-localized SIN proteins.

In this study, through RNA-Seq and genetic approaches, we identified a new positive septation regulator, MztA, which acts antagonistically toward ParA to coordinately regulate SPB-localized SIN proteins SidB-MobA during septation in the filamentous fungus *A. nidulans*.

## Materials and methods

### Strains, media, and culture conditions

A list of *A. nidulans* strains and oligonucleotides used in this study is provided in Tables 1, 2. YAG (5 g/L Yeast extract + 1 mL/L Trace element + 20 g/L Glucose), YUU (YAG + 1.2 g/L Uridine + 1.1 g/L Uracil), MMPGR (50 mL/L Salt + 10 mL/L Glycerol + 0.5 mg/L Pyridoxine + 2.5 mg/L Riboflavin + 1 mL/L Trace element), MMPGRTUU (MMPGR + 1.2 g/L Uridine + 1.1 g/L Uracil + 11.9 g/L Threonine) and MMPPGRTUU (MMPGRUU with 100 mM p-Aminobenzoic acid). These media have been described in previous works (Gupta et al., 1976; Käfer, 1977). Growth conditions, crosses, and induction conditions for *alcA*(*p*)-driven expression were as described previously (Liu et al., 2003). Overexpression of tagged genes under the control of the *alcA* promoter were induced with threonine (Zhong et al., 2014). Standard DNA transformation procedures were used for *A. nidulans* (Osmani et al., 1988).

**Table 1:** *Aspergillus nidulans* strains used in this study.

**Strain**	***Genotype***	**Source**
TN02A7	*pyrG89; pyroA4;nkuA::argB2;riboB2;veA1*	Nayak et al., 2006
R21	*pabaA1;yA2*	FGSC
ZGB01	*ΔparA::pyrG; pyrG89; pyroA4; nkuA::argB2; riboB2;veA1*	Zhong et al., 2014
ZGB10	*alcA(p)-parA-pyr4;pyrG89;pyroA4;nkuA::argB2; riboB2;veA1*	Zhong et al., 2014
JPA01	*ΔmztA::pyroA;alcA(p)-parA-pyr4; pyrG89;pyroA4;nkuA::argB2;riboB2;veA1;*	this study
JPA02	*ΔmztA::pyroA; pyrG89; pyroA4;nkuA::argB2;riboB2;veA1*	this study
JPA03	*gpd(p)-mztA-pyroA4;alcA(p)-parA-pyr4;pyrG89; nkuA::argB2;riboB2;veA1;*	this study
JPA04	*ΔpdbA::pyroA;alcA(p)-parA-pyr4; pyrG89;pyroA4;nkuA::argB2;riboB2;veA1;*	this study
JPA05	*ΔpdbA::pyroA; pyrG89; pyroA4;nkuA::argB2;riboB2;veA1*	this study
JPA06	*gpd(p)-pdbA-pyroA4;alcA(p)-parA-pyr4; pyrG89;nkuA::argB2;riboB2;veA1;*	this study
JPA07	*ΔpdaA::pyroA;alcA(p)-parA-pyr4; pyrG89;pyroA4;nkuA::argB2;riboB2;veA1;*	this study
JPA08	*ΔpdaA::pyroA; pyrG89; pyroA4;nkuA::argB2;riboB2;veA1*	this study
JPA09	*gpd(p)::pdaA::pyroA4;alcA(p)-parA-pyr4; pyrG89;nkuA::argB2;riboB2;veA1;*	this study
JPA10	*ΔpamA::pyroA;alcA(p)-parA-pyr4; pyrG89;pyroA4;nkuA::argB2;riboB2;veA1;*	this study
JPA11	*ΔpamA::pyroA; pyrG89; pyroA4;nkuA::argB2;riboB2,veA1*	this study
JPA12	*gpd(p)-pamA-pyroA4;alcA(p)-parA-pyr4; pyrG89;nkuA::argB2;riboB2;veA1;*	this study
JPA13	*Δcdc5A::pyroA;alcA(p)-parA-pyr4; pyrG89;pyroA4;nkuA::argB2;riboB2;veA1;*	this study
JPA14	*Δcdc5A::pyroA; pyrG89; pyroA4;nkuA::argB2;riboB2;veA1*	this study
JPA15	*gpd(p)-cdc5A-pyroA4;alcA(p)-parA-pyr4;pyrG89;nkuA::argB2;riboB2;veA1;*	this study
JPA16	*mztA-pyrG; pyrG89; pyroA4; nkuA::argB2;riboB2;veA1*	this study
JPA17	*mztA-pyrG; ΔmztA::pyroA;pyrG89;pyroA4;nkuA::argB2;riboB2;veA1*	this study
JPA18	*mztA-pyroA; alcA(p)-parA-pyr4; pyrG89;pyroA4; nkuA::argB2;riboB2; veA1;*	this study
JPA19	*gpd(p)-mztA;pQa-pyroA;pyrG89;pyroA4; nkuA::argB2;riboB2;veA1*	this study
JPA20	*gpd(p)-mztA;pJH37; riboB2;veA1 ΔAnmztA::pyroA;pyrG89;pyroA4;nkuA::argB2;*	this study
JPA21	*ΔparA::pyrG;ΔmztA::pyroA;pyrG89;pyroA4;nkuA::argB2; riboB2;veA1*	this study
JPA22	*alc(p)-mobA-pyr4;pabaA; yA2;veA1;*	this study
JPA23	*alc(p)-mobA-pyr4;gpd(p)-mztA;pQa-pyroA;pabaA; yA2;pyrG89;pyroA4; nkuA::argB2;riboB2;veA1*	this study
JPA24	*alc(p)-mobA-pyr4;alcA(p)-parA-pyr4;pabaA;yA2; pyrG89;pyroA4;nkuA::argB2;riboB2; veA1;*	this study
JPA25	*alc(p)-mobA-pyr4;gpd(p)-mztA;alcA(p)-parA-pyr4;pQa-pyroA;pabaA;yA2; pyrG89;pyroA4;nkuA::argB2;riboB2; veA1;*	this study

**Table 2:** Primers used in this study.

**Primer name**	**DNA sequence 5′-3′**
RT-actin-5′	TCTTCCAGCCCAGCGTTCT
RT-actin-3′	GGGCGGTGATTTCCTTCTG
RT-mztA-5′	CTTCACATACTGATTGCTTC
RT-mztA-3′	CGGGGTTGACTCCATT
RT-pdbA-5′	CGAAGTCGCTGCTACTATCC
RT-pdbA-3′	TATGCGTGCTCCATCCTG
RT-pdaA-5′	CGCCCACATTAGAGCAGT
RT-pdaA-3′	TCATAGCCTCGTAAACAGC
RT-pamA-5′	TTATCTCCGTCATTGGCTTTG
RT-pamA-3′	CGATGTTCTTTCCGTGCTG
RT-cdc5A-5′	ATGGGTGCCTTTGGTGAT
RT-cdc5A-3′	GAGCCGTCTGGTGAGTTTG
pyroA-5′	TTGGCGGGTAAGTCAGATAATAG
pyroA-3′	CTGACTTGACGCTTTCTCTTGG
P1-mztA	GGAGCAGACGGACAGATT
P2-mztA	CGATGGTATGGGAATGGT
P3-mztA	CTATTATCTGACTTACCCGCCAACTTGGCTTCGCAGAGTGT
P4-mztA	CCAAGAGAAAGCGTCAAGTCAGCCATTTACTCGGTTTCC
P5-mztA	GAAGACAGTATGCCTGAAAC
P6-mztA	CCATCCGCATTCTTCAC
P1-pdbA	GGAGCAATGAGCCAGAA
P2-pdbA	GCCACGAAAGACATAGAAC
P3-pdbA	CTATTATCTGACTTACCCGCCAACGAGGGTAGGAGAAAGGAG
P4-pdbA	CCAAGAGAAAGCGTCAAGTCAGTTGGACAGAGGGATTGAG
P5-pdbA	GCTGAAGCCTTGGGTAAT
P6-pdbA	ATTGGATGTGGAGGGAAA
P1-pdaA	GGAATAGGCGTATCGTTT
P2-pdaA	CTAAGCAAGGGACAGAAGC
P3-pdaA	CTATTATCTGACTTACCCGCCAAGATCTTTCTTAGGGTTGAGG
P4-pdaA	CCAAGAGAAAGCGTCAAGTCAGATACCGACAACATCGTCAAGG
P5-pdaA	CCAATGAACCCACTGAGAA
P6-pdaA	CCTGCCGAGTCGTCTAATCC
P1-pamA	GATACATCCAGCAGACCACT
P2-pamA	ATCCACAAGCACCAGAACC
P3-pamA	CTATTATCTGACTTACCCGCCAACAAACGCTTGTCAGCACTA
P4-pamA	CCAAGAGAAAGCGTCAAGTCAGAGTCGTTGGCTGTGGGTAT
P5-pamA	CAGCCAAGGAAAGCAACTAA
P6-pamA	GGTTGGGAAGAAGAAGGT
P1-cdc5A	GGGAACGGCGAAACCAAC
P2-cdc5A	GTAAACCACGCAGGGACG
P3-cdc5A	CTATTATCTGACTTACCCGCCAACAAACCCAGACTGACGAAAT
P4-cdc5A	CCAAGAGAAAGCGTCAAGTCAGGGCATAAAGGAGATACACG
P5-cdc5A	AGGGCACTCGCTGAAGAA
P6-cdc5A	CTTACAGCGACGGAACTG
NotI-pyroA-5′	ATTTGCGGCCGCTTTATTGGCGGGTAAGTCAGATAATAG
SpeI-pyroA-3′	GGACTAGTCCCTGACTTGACGCTTTCTCTT GG
mztA-ClaI-5′	CCATCGATGGATGCTAAGCTTGCTTCACAT
mztA-ClaI-3′	CCATCGATGGTCACTCTGGAGACTCATTCG
pdbA-ClaI-5′	CCATCGATGGATGGTACCCATTCCACGAG
pdbA-ClaI-3′	CCATCGATGGTCAATACTCAAGTGTTCGCT
pdaA-SmaI-5′	TCCCCCGGGGGA ATGACTCCTCTAATATATCTACCGGG
pdaA-SmaI-3′	TCCCCCGGGGGACTACGAGTCCAGTCCTTTGACTC
pamA-ClaI-5′	CCATCGATGGATGGCTAAGGAAACTAGATTC
pamA-ClaI-3′	CCATCGATGGCTAGTCCTTCTTCAAATCAACA
cdc5A-ClaI-5′	CCATCGATGG ATGACAGAACCTCGCCGGC
cdc5A-ClaI-3′	CCATCGATGGCTCAATACCCAAACGTCTCCTGC
gpdA-5′	GCATGCGGAGAGACGGACG
mztA-3′	CTCTGGAGACTCATTCG
pdbA-3′	GGCACCGACACCAAAGT
pdaA-3′	CCTCCTCCGTAACCTCA
pamA-3′	CTAGTCCTTCTTCAAATCAACA
cdc5A-3′	CTCAATACCCAAACGTCTCCTGC
mztA-5′	ATGCTAAGCTTGCTTCACATAC
pdbA-5′	CCTCCCACCCTCCAGTA
pdaA-5′	GCGACCCAGATACCACA
pamA-5′	ATGGCTAAGGAAACTAGATTC
cdc5A-5′	ATGACAGAACCTCGCCGGC
n-mztA-5′	ATCTGCCCTTATCCACCC
n-mztA-3′	AAGAGCATTGTTTGAGGCAAAGCTCCTCACCATCCC
n-mztA-2-3′	CTATTATCTGACTTACCCGCCAAAAAGCTCCTCACCATCCC
Af-pyrG-5′	GCCTCAAACAATGCTCTT
Af-pyrG-3′	CTGATGCGTGATGCCAAG
P1-gpd-mztA	ACCGTCATCACCGAAAC
P2-gpd-mztA	GCATGCGGAGAGACGGACG
P3-gpd-mztA	GTATGTGAAGCAAGCTTAGCATCAACGACCTTGTCAACCC
mztA-up	ATGCTAAGCTTGCTTCACATAC
mztA-down	TCACTCTGGAGACTCATTCG
P4-riboB-5′	CGAATGAGTCTCCAGAGTGATCCATCGCTTGCCCTTTCTG
P5-riboB	GCACCCGCTCTTTCACCACC
P6-riboB-3′	GGGTGATTGGAACTAACTGGAC
Probe-mztA	GGGCATCGGGGTTGACTCCATTCTCAATCAAAGACACGCAAAGAGAGAGCTCTGTCCG
Probe-parA	AGTCCGTATACCCTCCGACATATCCTTCGGGGTTGTTTGGAGGCGATCGAAGACATG
Diag-mztA-5′	TTTGCGTGTCTTTGATTG
Diag-mztA-3′	TCCGTCCTCAAGTCTTTG
Diag-parA-5′	ATGAAGGGTTTCAGACAGAGA
Diag-parA-3′	GTCTTCGGTTAATGTCGAGTC
mobA-NotI-5′	CGGCTAGCCGATGGCTTCATTCAT
mobA-XbaI-3′	GCTCTAGATATCCACCAGTCAG
Diag-GFP-5′	CACCGACCTACACGCTATG
Diag-mobA-3′	GGTAATGGTGGCAATAGATG

### Quantitative real-time PCR analysis

Conidia of *parA*-overexpressing mutant and the parental wild type (TN02A7) were cultured in MMPGRTUU for 18 h at 37°C with a rotary shaker at 220 rpm and then the collected dried hyphae were pulverized to a fine powder in the presence of liquid nitrogen. The total RNA was extracted using TRIzol (Roche) following the manufacturer's instructions. The samples were treated with DNase I (TaKaRa), and cDNA was generated using an iScript Select cDNA synthesis kit (Bio-Rad). Real-time PCR was performed using an ABI one-step fast thermocycler (Applied Biosystems), and the reaction products were detected with SYBR green (TaKaRa). PCR was accomplished by a 10-min denaturation step at 95°C followed by 40 cycles of 95°C-30 s, 60°C-30 s, 72°C-30 s. Transcript levels were calculated by the comparative C_T_ method and normalized against the expression of the actin gene in *A. nidulans* (Alam et al., 2012; Long et al., 2016). Primer information is provided in Table 2.

### Construction of mutant strains

For construction of the OE::*parA* ^Δ*mztA*^ and the Δ*mztA* mutants, the *A. nidulans AnpyroA* gene, which is required for biosynthesis of pyridoxine, was amplified from plasmid pQa-pyroA with the primer pairs pyroA-5′/ pyroA-3′, and used as a selectable nutritional marker for fungal transformation. The upstream and downstream regions of the *mztA* gene were amplified with primers P1-mztA/P3-mztA and P4-mztA/P6-mztA from the *A. nidulans* TN02A7 genomic DNA, respectively. Purified linearized upstream and downstream DNA fragments plus the *pyroA* gene fragment were mixed and used in a double joint PCR with primers P2-mztA/P5-mztA (Yu et al., 2004). The resulting fusion fragment was then cloned into the pEasy-Blunt Zero (TransGen Biotech) vector for generating plasmid pJP01. Finally, the pJP01 plasmid was respectively transformed into the OE::*parA* strain and the parental wild-type strain to generate strains OE::*parA* ^Δ*mztA*^ (JPA01) and Δ*mztA* (JPA02), A similar strategy was used to construct the OE::*parA*^Δ*pdbA*or Δ*pdaA*or Δ*pamA*or Δ*cdc*5*A*^ and Δ*pdbA* (Δ*pdaA* or Δ*pamA* or Δ*cdc5A*) strains. Briefly, as shown in Figure S1A, upstream and downstream regions of these five genes were amplified from gDNA with primer pairs P1/P3 and P4/P6. Each two regions were then fused to the *AnpyroA* gene cassette with the nested primer pair P2/P5 and cloned into plasmid pEasy-Blunt Zero. The resulting plasmids were further transformed into OE::*parA* strain and the parental wild type (TN02A7) to generate related strains. Oligonucleotides used in this study are listed in Table 2. To construct the Δ*parA*Δ*mztA* mutant, the Δ*mztA* mutant was crossed with the Δ*parA* mutant. All progeny were screened according to a standard protocol (Todd et al., 2007).

For constructions of the overexpressed aforementioned five genes in the background of *parA*-overexpressing, the *A. nidulans AnpyroA* gene, a selectable nutritional marker, was amplified with primer pair NotI-pyroA-5′/SpeI-pyroA-3′. Then the *AnpyroA* fragment was cloned into the reconstitute vector pBARGPE1 using the ClonExpress II One Step Cloning Kit (VazymeTM, C112-02), this strain was designated pBARGPE1-1, which contains the *AngpdA* promoter. Using gDNA as a template, the ORF fragments of those five genes were amplified with primer pairs mztA-ClaI-5′/mztA-ClaI-3′, pdbA-ClaI-5′/pdbA -ClaI-3′, pdbA-SmaI-5′/pdbA-SmaI-3′, pamA-ClaI-5′/ pamA-ClaI-3′, cdc5A-ClaI-5′/cdc5A-ClaI-3′, respectively, and subsequently were cloned into pBARGPE1-1. The resulting relevant plasmids were transformed into the OE::*parA* strain.

For generation of strains WT^*mztA*^, Δ*mztA*^*mztA*^, OE::*parA*^*mztA*^, the parental wild-type *mztA* gene driven by the endogenous promoter was introduced into WT, Δ*mztA*, OE::*parA*, respectively. An 1876-bp *AfpyrG* fragment was amplified with primer pairs Af-pyrG-5′/Af-pyrG-3′ from plasmid pXDRFP4. The 1759-bp *AnpyroA* gene was amplified with primer pair pyroA-5′/pyroA-3′ from plasmid pQa-pyroA. *AfpyrG* and *AnpyroA* were chosen as two selectable nutritional markers, so fragments of *mztA* gene with endogenous promoter was amplified from the *A. nidulans* TN02A7 genomic DNA with primers n-mztA-5′/n-mztA-3′ and n-mztA-5′/n-mztA-2-3′, respectively, referred as fragments 1 and 2. Then the fragment 1 was fused with *AfpyrG* and PCR cassette was cloned into plasmid pEasy-Blunt Zero and transformed into strain TN02A7, Δ*mztA* to generate WT^*mztA*^, Δ*mztA*^*mztA*^. Fragment 2 subsequently was fused with *AnpyroA* and the resulting fusion product was cloned into plasmid pEasy-Blunt Zero then transformed into strain OE::*parA* to obtain the OE::*parA*^*mztA*^. For WT^OE::mztA^, Δ*mztA*^OE::mztA^ construction, the plasmid containing *mztA* gene controlled by *gpdA* promoter and selective markers *pyroA* or *riboB* genes were co-transformed into the indicated mutants.

To generate an *alcA(p)-gfp-mobA* fusion construct, a 594-bp fragment of *mobA* was amplified from TN02A7 genomic DNA by use of primers mobA-NotI-5′ /mobA-XbaI-3′ (see Table 2) and then *mobA* was cloned into the corresponding sites of pLB01, yielding pLB-mobA (Liu et al., 2003). This plasmid was transformed into the receipt strain R21. Homologous recombination of this plasmid into the *mobA* locus should result in an N-terminal green fluorescent protein (GFP) fusion with the product of the entire *mobA* gene under the control of the *alcA* promoter. This strain was referred as *alc(p)-mobA-*GFP. For generation of OE::*parA*^*mobA*^^−GFP^, OE::*mztA*^*mobA*^^−GFP^ and OE::*parA* OE::*mztA*^*mobA*^^−GFP^ strains, *alc(p)-mobA-*GFP was crossed with the OE::*parA*, OE::*mztA* and OE::*parA* OE::*mztA* mutants, respectively.

### Microscopy and image processing

To visualize the process of septation, conidia of indicated strains were incubated in indicated liquid medium on sterile glass coverslips, respectively, at related temperatures prior to observation under a microscope. The signal of septum was observed in live cells by placing the coverslips on a glass slide. Then removed the media from coverslips and washed three times by phosphate buffered saline (PBS). After that, 4% paraformaldehyde (Polyscience, Warrington, PA) was used to fix the cells and Calcofluor white (CFW) (Sigma-Aldrich, St. Louis, MO) was added for septum and chitin staining for keeping in dark for 10 min. After staining, the staining solution was removed and washed with PBS for three times. Finally, above coverslips were placed on a glass slide and sealed with nail oil. Microscopic images of cells were collected with a Zeiss Axio Imager A1 microscope (Zeiss, Jena, Germany). These images were then collected and analyzed with a Sensicam QE cooled digital camera system (Cooke Corporation, Germany) with the MetaMorph/MetaFluor combination software package (Universal Imaging, West Chester, PA), and the results were assembled in Adobe Photoshop 7.0 (Adobe, San Jose, CA). A similar approach was taken to visualize the localization of MobA-GFP in related strains. 4′,6-diamidino-2-phenylindole (DAPI) (Sigma-Aldrich, St. Louis, MO) was used to stain DNA.

### RNA isolation for northern analysis

All test mutants were grown in the liquid medium-MMPGRTUU shaken on a rotary shaker at 220 rpm at 37°C. G7 is referred as the harvest detection time-point when conidia were cultured for 7 h. V12 and V18 represent the vigorous hyphal growth time-point when conidia were cultured for 12 or 18 h. S24 presents the sporulation time-point when conidia were cultured for 18 h and then the liquid cultures were transferred to the solid culture plate for 24 h. Pulverized the mycelia of those strains to fine powder in the presence of liquid nitrogen. RNA purification and Northern blot analysis were performed as described. Briefly, the total RNA was extracted using TRIzol (Roche) following the manufacturer's instructions. 10 μg of RNA was used for electrophoresis on 1.1% formaldehyde agarose gels and subsequently blotted onto a nylon membrane (Bio-Rad). Probed are labeled with DIG (digoxigenin)-labeled oligonucleotide probes complementary to the mRNA of *mztA* or *parA*. Primer information is provided in Table 2. The blots are pre-hybridized with high-SDS for at least 5–7 h at 42°C. Late in the afternoon the DIG-probes are heated up to 65°C for 10 min and the high-SDS solution in the tube containing the membrane was exchanged with the probe. This is then further incubated overnight at 42°C. Finally, signals of the RNA bands were showed by using an CSPD(Sigma) (Misslinger et al., 2017).

## Results

### Identification for the suppressors of ParA in septation and conidiation

**Figure 1:**
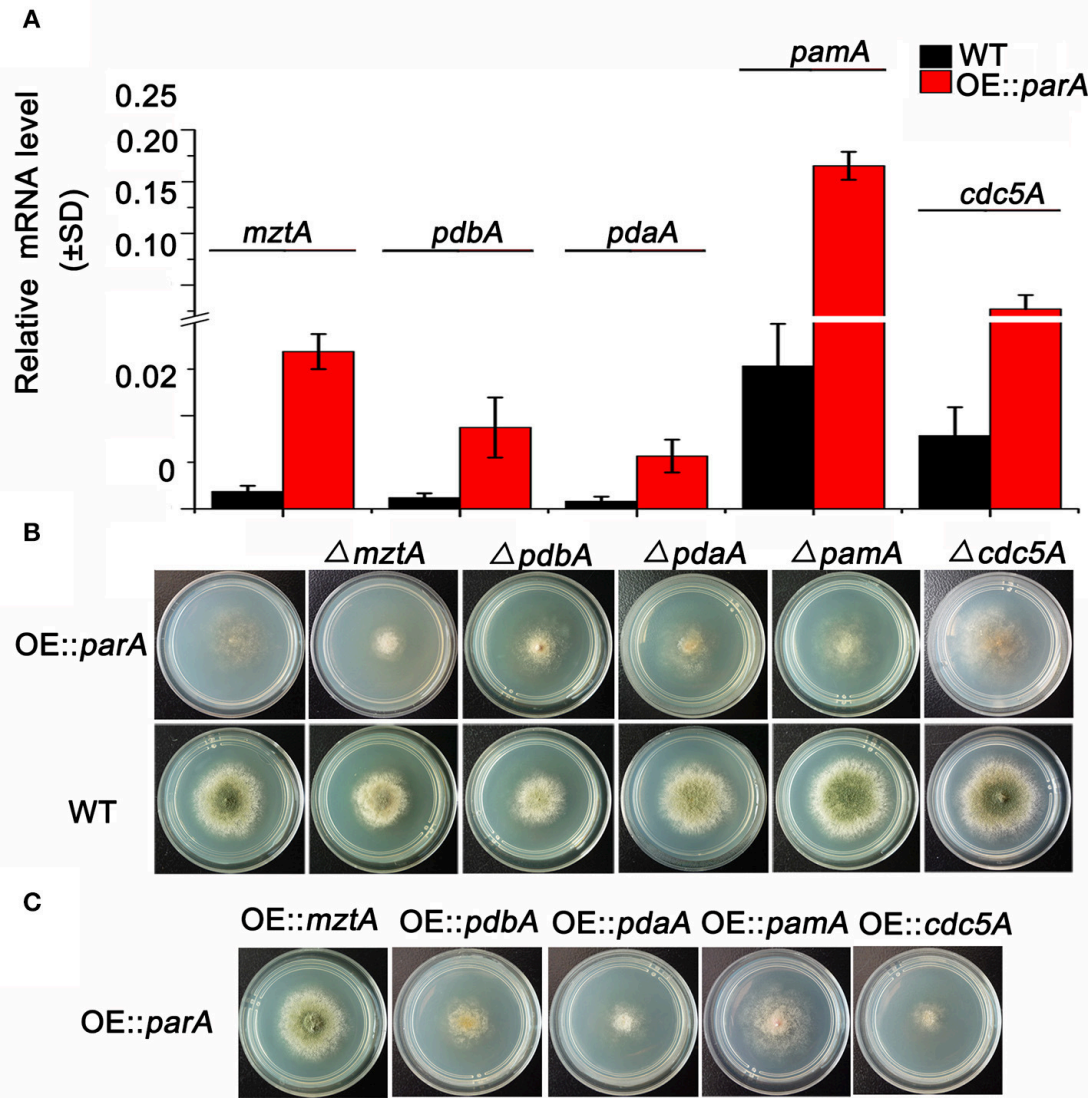
Identification of suppressors of *parA* in septation and conidiation. **(A)** The relative mRNA levels of *mztA, pdbA, pdaA, pamA*, and *cdc5A*, respectively, using real-time RT-PCR assays in the *parA*-overexpressing mutant and parental wild type (TN02A7). Those two strains cultured in liquid MMPGRTUU medium at 37°C at 220 rpm for 18 h. The measured quantity of the mRNA in each of the treated samples was normalized using C _T_ values obtained for the internal reference actin (AN3696.4). **(B)** Phenotypic characterization of *mztA, pdbA, pdaA, pamA*, and *cdc5A* deletion mutants in the background of the *parA*-overexpressing mutant and the parental wild type (TN02A7), respectively. All the strains were cultured on solid MMPGRTUU medium for 2.5 days. **(C)** Colony morphologies of the overexpressed mutants for *mztA, pdbA, pdaA, pamA* and *cdc5A* genes in the background of the *parA*-overexpressing mutant. All the strains were cultured on solid MMPGRTUU medium for 2.5 days.

**Figure 2:**
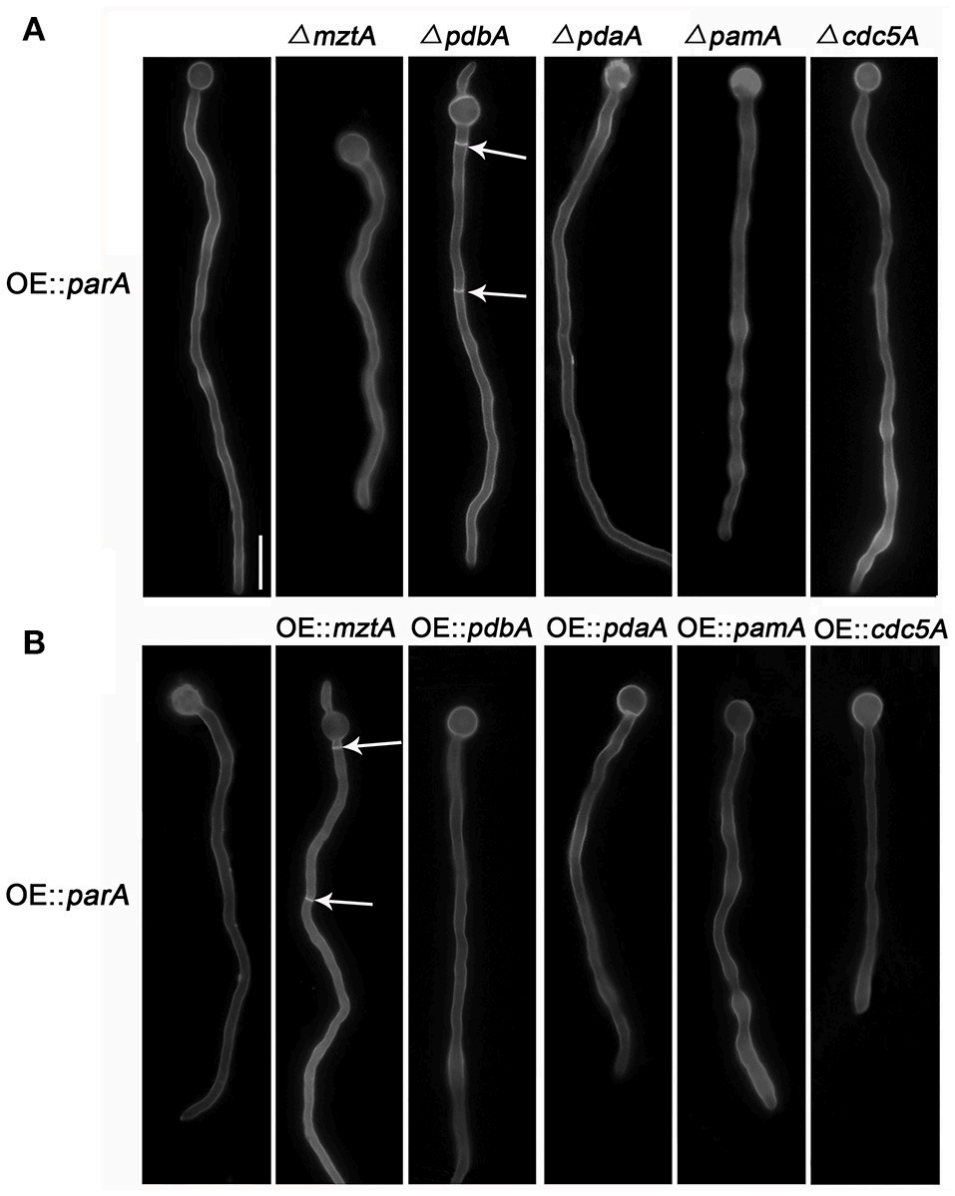
Hyphal morphologies of the *mztA, pdbA, pdaA, pamA*, and *cdc5A* deletions **(A)** and their overexpression **(B)** in the background of the *parA*-overexpressing mutant cultured in liquid MMPGRTUU media and stained with Calcofluor white (septa). All the strains were cultured at 37°C for 11 h. Arrows indicate the locations of septa. Bars, 10 μm.

Our previous data confirmed that ParA, a regulatory subunit of protein phosphatase 2A (PP2A), is a negative regulator of septation since deletion of *parA* causes a hyperseptation phenotype while overexpressing *parA* results in abolished septum formation in hyphal cells (Zhong et al., 2014). To further address how overexpressing *parA* causes completely abolished septation, RNA-Seq analysis was performed in the *parA*-overexpressing mutant compared to that of its parental wild-type strain (TN02A7). A total of 551 mRNAs had an altered abundance in the *parA*-overexpressing mutant compared with the parental wild-type strain when using a 2-fold change as a cutoff. Among them 453 genes were up-regulated and 98 genes were down-regulated. To analyze the affected gene expression induced by overexpressing *parA*, we selected candidate genes based on a putative septation-related function in *S. pombe* as well as having a ratio>5 for their fold change. Based on these two criteria, five candidate genes were obtained with up-regulation of 9.8-, 9.1-, 8.9-, 7.9-, and 7.6-fold the OE::*parA* mutant. As queries in the NCBI and CADRE databases in *A. nidulans*, those five genes are referred as *mztA* (AN1361.4, Accession:Q5BDL9.1), *pdbA* (AN8559.4,Accession: CBF80798.1)*, pdaA* (AN1726.4,Accession: CBF85442.1), *pamA* (AN3493.4,Accession: CBF76042.1) and *cdc5A* (AN7174.4,Accession: CBF78928.1), respectively. To verify that overexpressing *parA* caused the changed expression of those five genes as seen in the RNA-Seq data, a real-time PCR was further carried out to visualize the mRNA abundance level of these five genes in the OE::*parA* strain. As shown in Figure 1A, the expression level of *mztA, pdbA, pdaA, pamA* and *cdc5A* was increased 8.03-, 7.30-, 7.00-, 7.02-, and 4.88-fold compared to that of its reference strain, respectively, which was comparable to that of RNA-Seq data for some content. Therefore, we hypothesize that one of reasons for completely abolished septation in the *parA-*overexpressing strain might be the result of the overexpression of the aforementioned five genes. To check whether deleting those five genes could rescue the septation defect of the OE::*parA* strain, we constructed *mztA-, pdbA-, pdaA-, pamA-*, and *cdc5A-*null mutants in the background of OE::*parA* and the parental wild-type strains. Diagnostic PCR analysis showed that those deletion mutants had the correct insertion of the *AnpyroA* disruption cassette in the relative gene coding sequence loci, and no original ORF of those five genes could be detected, indicating that the relative genes were fully deleted in the background of OE::*parA* and the parental wild-type strains (Figure S1). As shown in the upper panel of Figure 1B, none of those deletions could suppress the nearly aconidial colony morphology in the *parA*-overexpressing mutant. Likewise, under the liquid culture conditions, four of them could not rescue the septation defect eourourxcept for *pdbA* deletion, which caused a septum restoration in the background of the OE::*parA* mutant (Figure 2A). These data suggest that completely abolished septation in the *parA-*overexpressing strain might not directly result from the overexpression of the aforementioned four genes. While overexpressed *pdbA* may be related to the defective septation in the *parA*-overexpressing strain. In addition, Δ*mztA* and Δ*pdbA* exhibited a slightly attenuated conidiaton, plus decreased colony diameters of approximately 70 and 50%, respectively, of that of its parental wild type (Figure 1B), while the *pdaA* deletion mutant only showed decreased conidiation with almost normal colony size. In comparison, Δ*pamA* and Δ*cdc5A* showed WT-like colony phenotypes.

In contrast, we next wondered that whether overexpressing those five genes could rescue the sick morphology of the *parA*-overexpressing mutant. We then over-expressed them by an *AngpdA*-driven constitutive promoter in the background of the *parA*-overexpressing strain. Diagnostic PCR revealed those related strains that were constructed successfully (Figure S2). Notably, over-expression of *mztA* significantly restored the OE::*parA* mutant to a phenotype with WT-like conidiation and radial hyphal growth as shown in Figure 1C. In comparison, over-expression of the other four genes could not rescue the aconidial colony to the wild-type phenotype. Consistently, liquid cultural observation demonstrated that OE::*mztA* could rescue the septation defect of over-expressed *parA*, while there were no obvious differences for septation between the other four over-expression strains and its parental wild-type strain (Figure 2B). Therefore, those results suggest that *mztA* works as a suppressor of *parA* in septation and conidiation in *A. nidulans*.

### Septation and conidiation defects induced by overexpressing *parA* are rescued by overexpressing *mztA* in a dose-dependent way

**Figure 3:**
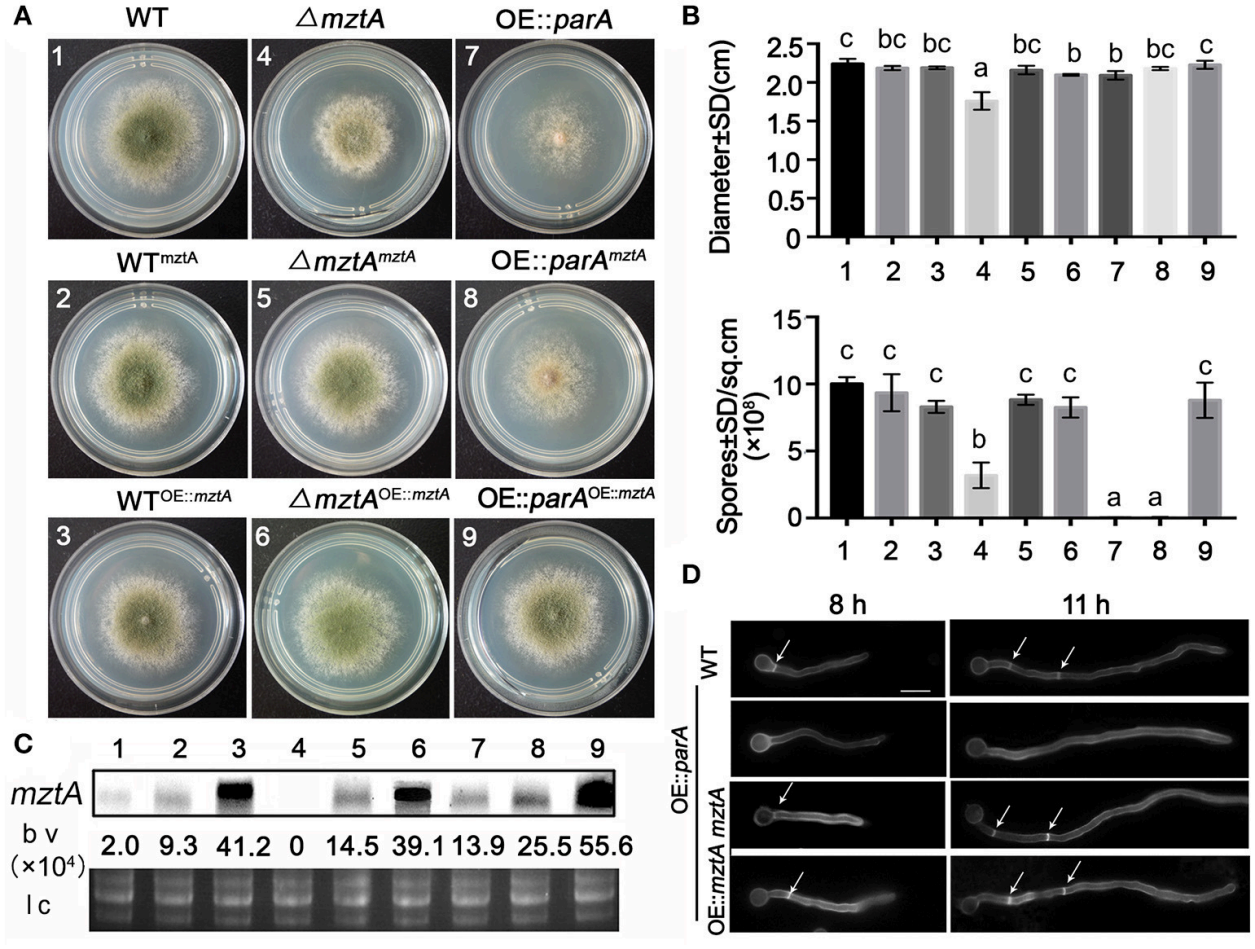
Septation and conidiation defects induced by overexpressed *parA* are rescued by overexpressed *mztA* in a dose-dependent manner. **(A)** The colony morphology of the overexpressed *mztA* mutant under the control of its endogenous promoter or a constitutive promoter “*Angpd(p)*” in the parental wild type (TN02A7), Δ*mztA* and *parA*-overexpressing strains, respectively. The superscript “*mztA*” represent the overexpression *mztA* controlled by the endogenous promoter. The superscript “OE::*mztA*” represents the overexpression of *mztA* controlled by the “*Angpd(p)*” promoter. **(B)** Quantitative data for the diameter and quantitative number of spores for the strains shown in panel A. The number under the histogram indicates the WT, WT^*mztA*^, WT^OE::mztA^,Δ*mztA*,Δ*mztA*^*mztA*^, Δ*mztA*^OE::mztA^, OE::*parA*, OE::*parA*^*mztA*^, and OE::*parA*^OE::mztA^. Error bars represent the standard deviation from three replicates. Different lowercase letters on the bars of each group represent significant differences among the strains. *P* < 0.05. **(C)** Expression level of *mztA* mRNA was detected by Northern blotting analysis with RNA extracted from the strains shown in panel A. The capital letters,1-9, indicate the WT, WT^*mztA*^, WT^OE::mztA^,Δ*mztA*,Δ*mztA*^*mztA*^, Δ*mztA*^OE::mztA^, OE::*parA*, OE::*parA*^*mztA*^, and OE::*parA*^OE::mztA^. All the strains cultured in liquid MMPGRTUU at 37°C 220 rpm for 18 h. Equal loading of the RNA samples were evaluated by 28 S and 18 S RNA bands. Numbers below the blots denoted the relative density of each band normalized as calculated using ImageJ. b v: band volume; l c: loading control. **(D)** Comparison of the septum formation in hyphal cells between the parental wild type (TN02A7), OE::*parA*, OE::*parA*^*mztA*^, and OE::*parA*^OE::mztA^. All the strains were cultured in liquid MMPGRTUU medium for 8 h and 11 h, respectively. Arrows indicate the locations of septa. Bars, 10 μm.

The abovementioned phenomena led us to question whether the restoration of the defective phenotype in the OE::*parA* mutant by overexpressing *mztA* is in a dose-dependent. To test this hypothesis, we constructed six complementation strains by introducing the parental wild-type *mztA* gene driven by the endogenous promoter and the constitutive promoter (*gpdA*) into Δ*mztA*, OE::*parA* and the parental wild-type strains, respectively (Figure 3A). Diagnostic PCR analysis indicated that all the strains were constructed successfully (Figure S3). To further ensure that the expression level of *mztA* in these complementation strains was truly over-expressed, Northern blotting was used to detected the mRNA expressions and 28 and 18 S RNA as the loading control shown in Figure 3C. As expected, the mRNA abundance level of the *mztA* gene driven by an endogenous promoter in WT^*mztA*^, Δ*mztA*^*mztA*^ and OE::*parA*^*mztA*^ were separately increased 4.65-, 7.25-, and 12.75-fold changes compared with the parental wild-type. Meanwhile, the *mztA* transcript level driven by the constitutive promoter (*gpdA*) in WT^OE::mztA^*, mztA*^OE::mztA^ and OE::*parA*^OE::mztA^ was up-regulated 20.6-, 19.55-, 27.8-fold times compared with the WT. These data indicated that introducing *mztA* into Δ*mztA*, OE::*parA* and the parental wild-type strains driven by the self-promoter and *AngpdA* promoter were truly resulted in the relative overexpression of *mztA*. As shown in Figures 3A,B, phenotypic analysis suggests that introducing either low copies or high copies of the *mztA* gene into WT, Δ*mztA* receipt strains had no obvious difference in colony size and conidia production compared to that of the parental wild type. Furthermore, the aconidial defect phenotype in the OE::*parA* mutant could be dramatically restored by over-expressed *mztA* gene under the control of the *gpdA* promoter to nearly the levels of the parental wild type strain and partially rescue of the conidiation defect was observed on the solid media of OE::*parA*^*mztA*^, which was controlled by native promoter (Figures 3A,B). Interestingly, in submerged liquid culture, we found that, in spite of low copies of the *mztA* gene, it could not completely restore the aconidiation defect of OE::*parA*, but it truely recovered the sepetation of OE::*parA* (Figure 3D). Collectively, the above results showed that restoration of the defective phenotypes of the OE::*parA* mutant showed a dose-dependent response to the over-expressed *mztA* gene.

### Reduced septa in Δ*mztA* are suppressed by deleted *parA*

**Figure 4:**
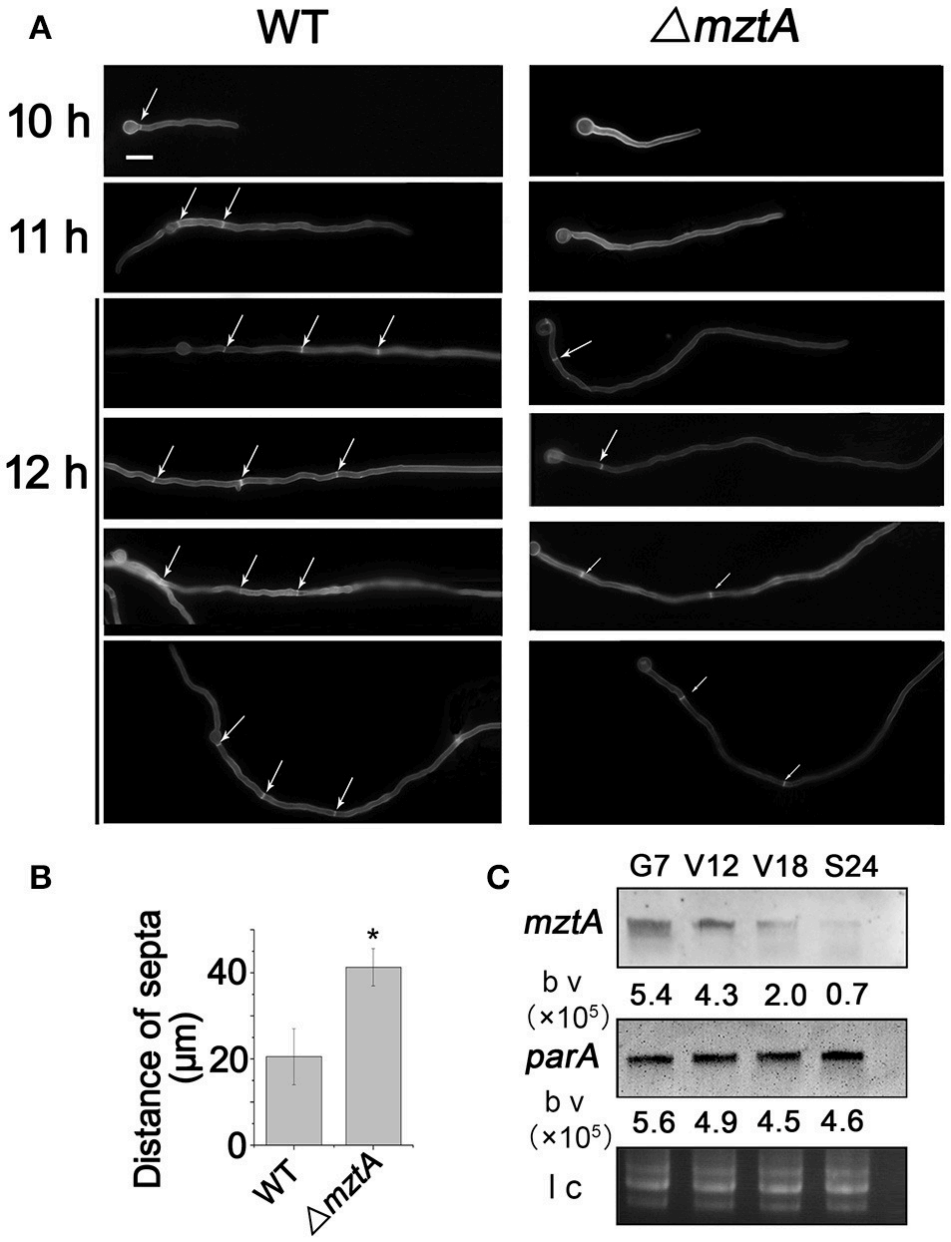
Abnormal distribution of the septa in the *mztA* deletion mutant and the mRNA levels for the *mztA* and *parA* genes during different development stages in the wild type (TN02A7). **(A)** Comparison of the septum distribution in hyphal cells between the parental wild type (TN02A7) and Δ*mztA* mutant. Those two strains were grown in MMPGRTUU medium at 37°C for 10,11, and 12 h. Septa were stained by Calcofluor white. Arrows indicate the locations of the septa. Bars, 10 μm. **(B)** Quantitative data for the septum distance of the WT and Δ*mztA* mutant under the same cultural condition and time. Statistical significance was determined by Student's test, with *P* < 0.05 (*). **(C)** Expression levels of *mztA* and *parA* mRNA were shown by Northern blotting analysis with RNA extracted from the wild-type strain (TN02A7) throughout the four developmental time-points. The capital letters, G7, V12, V18, and S24, indicate the germination time point (G7), the vigorous hyphal growth time point (V12 and V18) and the sporulation time point (S24), respectively. Equal loadings of RNA samples were evaluated by 28 and 18 S RNA bands (l c: loading control). Numbers below the blots denoted the relative density of each band normalized as calculated using ImageJ (b v: band volume).

**Figure 5:**
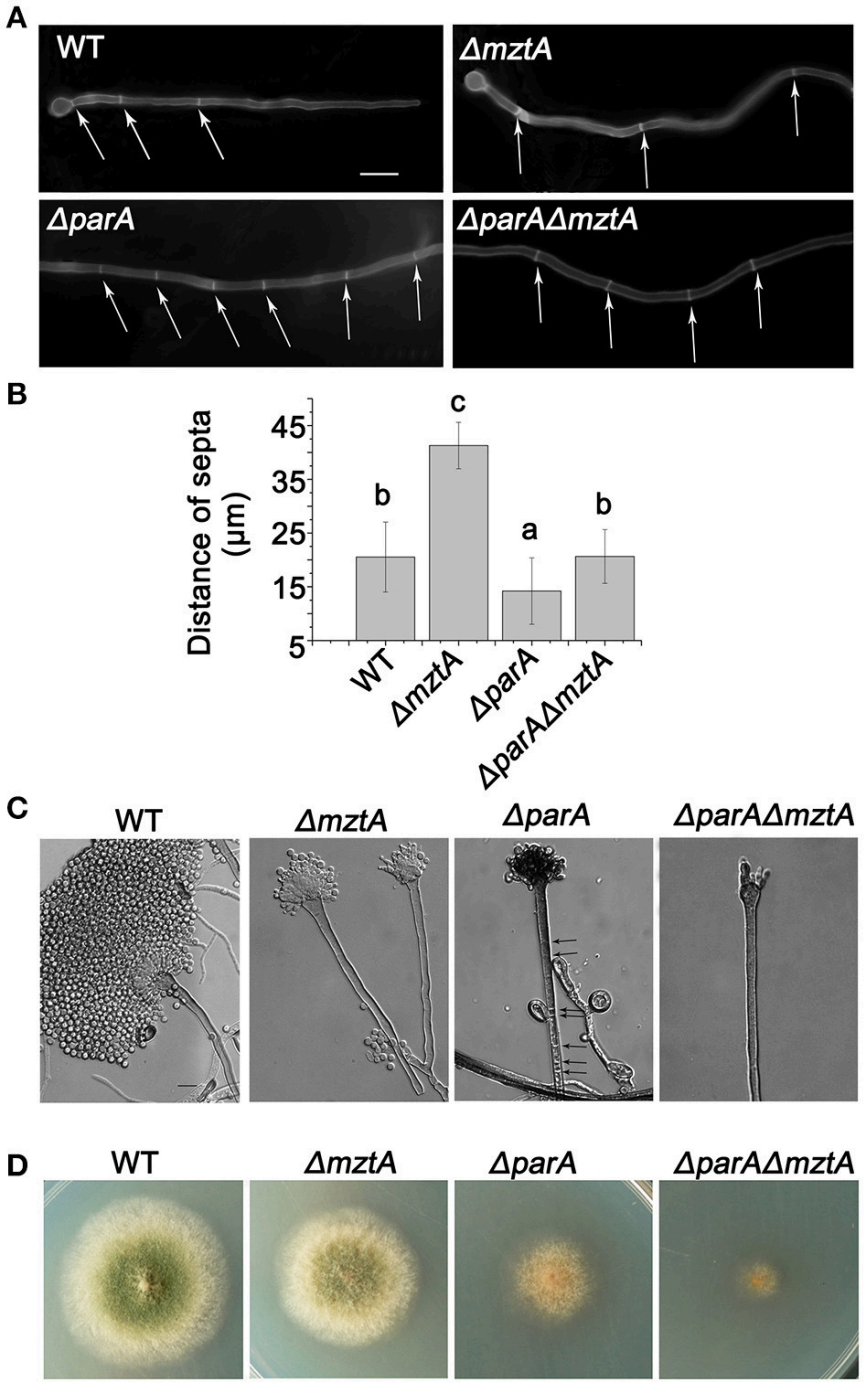
Reduced septa in Δ*mztA* are suppressed by deleted *parA*. **(A)** Comparison of the septum distribution in hyphal cells between the parental wild type (TN02A7), Δ*mztA*, Δ*parA* and Δ*parA*Δ*mztA*. All the strains were cultured in liquid MMPGRTUU medium at 37 °C for 12 h. Arrows indicate the locations of septa. Bars, 10 μm. **(B)** Quantitative data for the septum distance of the strains in **(A)**. Different lowercase letters on the bars of each group represent significant differences among strains. *P* < 0.05. **(C)** Conidiophores of the parent control strains (TN02A7), and the Δ*mztA*, Δ*parA*, and Δ*parA*Δ*mztA* mutants. Arrows indicate the locations of septa. Bars, 10 μm. **(D)** Colony morphologies of the parental wild type (TN02A7), and the Δ*mztA*, Δ*parA*, and Δ*parA*Δ*mztA* strains cultured on YUU at 37°C for 2 days.

With the aim of understanding the function of MztA during septation, the Δ*mztA* strain was analyzed under a dissecting microscope used to analyze the Calcofluor white staining shown in Figure 4A. When conidial spores were inoculated into liquid medium for 10, 11, and 12 h at 37°C, and hyphae were stained with CFW, the mutant displayed an abnormal reduced septa distribution in Δ*mztA* mature cells compared to that of the parental wild type (Figure 4A). The distance between the septa in the Δ*mztA* mutant was 41.29 ± 4.32 μm (*n* = 105) instead of the distance of 20.54 ± 6.51 μm (*n* = 105) seen in the parental wild type of the same hyphae length (Figure 4B). Thus, according to the average distance between the septa, we conclude that the Δ*mztA* mutant had a reduced or delayed septation defect phenotype. To better understand the relationship between *parA* and *mztA*, Northern blotting was carried out to visualize the mRNA abundance levels of these two genes in various developmental stages (Figure 4C). The 28 S and 18 S RNA were the loading control for Northern blotting. As a result, *parA* was abundantly expressed in all indicated stages, but *mztA* was strongly expressed in G7 and V12, and then weakly expressed in V18 and almost vanished in S24, suggesting that the expression pattern for *mztA* differs substantially from that of *parA*. Since the aforementioned data indicated that over-expressed *mztA* had a reverse effect on the overexpression of *parA* during septation, we further tested whether this suppressed phenotype for septation could be happen due to the double deletions of *parA* and *mztA*. By crossin*g* the Δ*parA* strain with the Δ*mztA* mutant as described in the Material and Methods section, a Δ*parA*Δ*mztA* double deletion strain was generated. Diagnostic PCR showed that both coding sequences of *parA* and *mztA* have not been detected in the double deletion mutant, suggesting that those strains were constructed successfully (Figure S4). As shown in Figure 5A, a microscopic study found that the single deletion of *parA* had a hyperseptation phenotype, while the *mztA* deletion mutant had a reduced-septa defect, and the double deletion strain showed almost normal septation formation compared to that of the parental wild type. Quantified analysis identified that the mature hyphal cells of Δ*parA* mutant were 14.21 ± 6.17 μm (*n* = 110), while the Δ*parA*Δ*mztA* double-deletion mutant had almost the normal septum formation with a septa distance of 20.65 ± 5.01 μm (*n* = 100; Figure 5B). These data suggest that the *mztA* deletion could suppress the hyperseptation of the Δ*parA* mutant. We also observed the morphology of the conidiophores in Δ*parA*, Δ*mztA*, Δ*parA*Δ*mztA*, and the parental wild type strains. *mztA* and *parA* single deletion strains could develop a few irregular metulae and phialides with some conidia, whereas in the *parA* and *mztA* double deletion mutant there were more severely defects of metulae, and most of the hyphal cells in the Δ*parA*Δ*mztA* mutant were unable to form these metulae structures. Interestingly, the *parA* deletion strain showed abnormal multiple septa in the stalk compared to the parental wild type strain having no any septa in the stalk, further indicating that *parA* is a negative regulator. In comparison, under the same culture condition, Δ*mztA* also showed a non-septum phenotype in the stalk. However, deletion of *mztA* could restore the abnormal multiple septa of the stalk in the deletion of *parA* to the wild-type non-septum phenotype (Figure 5C). Meanwhile, compared to the Δ*parA* or Δ*mztA* single mutant, the Δ*parA*Δ*mztA* double-deletion mutant showed more severe radical growth and conidiation defects with very tiny and aconidial colony but it was not the synthetic lethality phenotype (Figure 5D). All the data indicate that the *mztA* deletion was capable of suppressing the septation defect of the *parA* deletion and both *parA* and *mztA* are simultaneously required for hyphal growth, conidiation and septation.

### *parA* and *mztA* coordinately affect MobA localization

**Figure 6:**
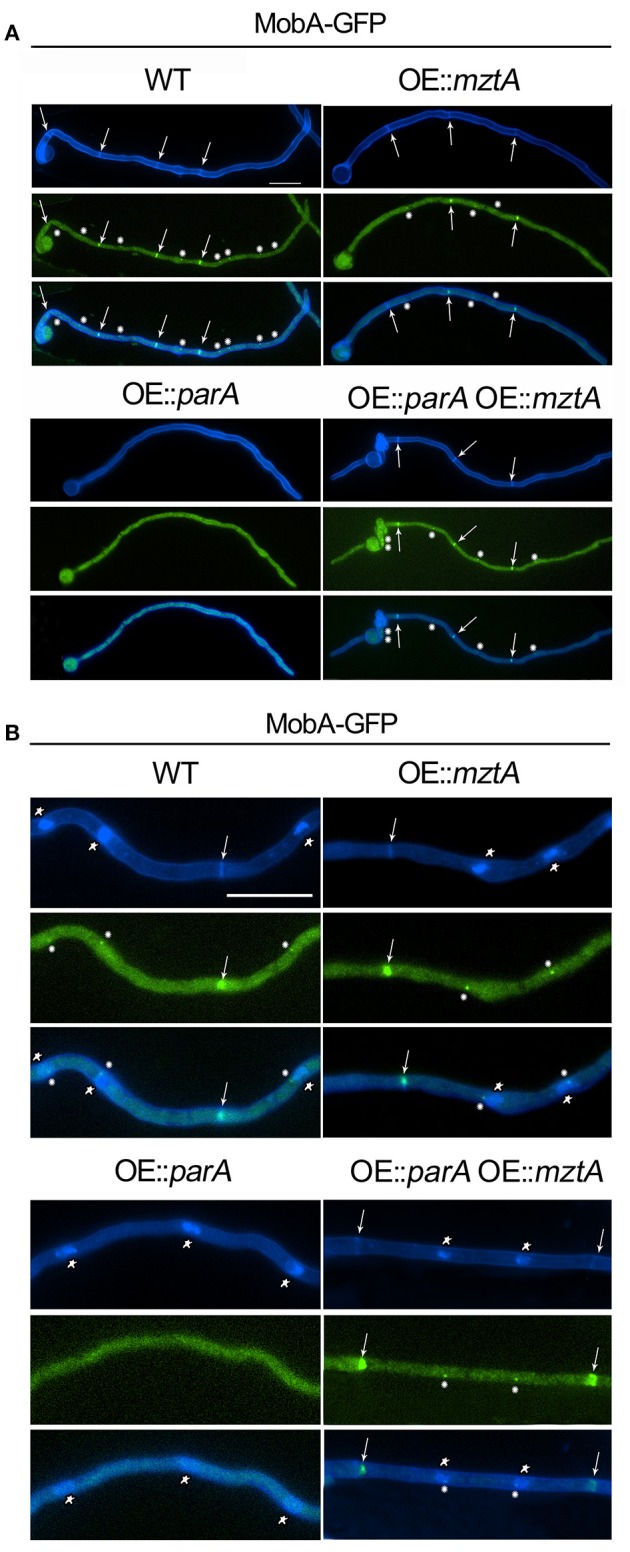
Localization patterns of GFP-tagged MobA in the parental wild type (R21), *mztA-*overexpressing, *parA-*overexpressing, and *mztA* and *parA-*double overexpressing mutants. Calcofluor white(CFW) was used to visualize the septa. **(A,B)** show different enlargement. All the strains were cultured in liquid medium MMPPGRTUU at 37°C for 12 h. Arrows indicate the location of septa, asterisk indicate the location of the spindle pole body (SPB) and polygon indicate the locations of nuclei. Bars, 10 μm.

According to the above results, we next asked how the function of *mztA* could suppress *parA* during septation. We speculated that *parA* and *mztA* may coordinately affect the recruiment of the components in the septation initiation network (SIN) during septation. We next found that the most well-known component of SIN, MobA, always appeared at the top of nuclei, which was known to be the position of spindle pole body (SPB). During septation, MobA localized to the septation site forming a ring and then accumulated gradually to the central region of the septation site. First, we constructed a conditional strain named *alc(p)-GFP-mobA*, in which the expression of *mobA* was able to be repressed by glucose on YAG medium, nonrepressed by glycerol on MMPGR, and induced by glycerol plus threonine on MMPGR. Diagnostic PCR indicated that the gene cassette was integrated into the predicted site in this conditional strain (Figure S5). Microscopic observation showed that GFP-MobA indeed localized at the SPB and septum site in the mature cell under induced condition. Based on the localization and function of MobA, we hypothesized that MobA might play an important role in ParA and MobA coordinately control of the SIN pathway. Thus, we generated strain JPA24, JPA25 and JPA26 which expressed the MobA protein as a GFP-tagged fusion protein on the background of OE::*mztA*, OE::*parA* and double overexpression *mztA* and *parA* strains by genetic-crossing *alc(p)-GFP-mobA* with relative strains, respectively. Microscopic observation showed that for the wild-type and the OE::*mztA* strains, during septation, MobA localized to the septation site forming a ring and then accumulated gradually to the central region of the septation site. However, GFP-MobA in the OE::*parA* mutant exhibited cytosol localization with no detectable signal at the SPB and the septum. Most interestingly, the double overexpression of *parA* and *mztA* strain showed a wild-type GFP-MobA signal localized at the SPB and the septation site to those in the *alc-GFP-mobA* mutant (Figures 6A,B). Our finding demonstrated that overexpressing *mztA* could rescued the abnormal localization of GFP-MobA induced by overexpressing *parA*.

## Discussion

Our previous data have confirmed that ParA, a regulatory subunit of protein phosphatase 2A (PP2A), is a negative regulator so that overexpression of PP2A-ParA causes complete abolished septation in *A. nidulans* (Zhong et al., 2014). MztA was reported to be a putative mitotic-spindle organizing protein and functioned in connecting the ring of γ-tubulin complex to the SPB (Masuda et al., 2013; Batzenschlager et al., 2014). Here, we found it is a new regulator of septation as a suppressor of PP2A-ParA in *A. nidulans*.

Using the RNA-Seq technique to systematically check the fold changes of mRNA expression, we found that overexpressing *parA* led to the up-regulation of a series of genes. According to the homolog information in *S. pombe* for the selected 5 genes, all indicated their functions might be related to septation. Therefore, by RT-PCR analysis, we further verified whether expression of these five genes was truly affected by overexpression of *parA*. As shown in Figure 1A, expression of the aforementioned five genes were significantly up-regulated in the overexpressed *parA* strain. However, deleting four of them (*mztA, pdaA, pamA, cdc5A*) could not rescue the conidiation and septation defects induced by overexpressing *parA*, suggesting completely abolished septation in the *parA-*overexpressing strain, which might not directly result from the overexpression of the aforementioned four genes. In comparison, the deletion of another gene, *pdbA*, a homologous gene of *pdb1* in *S. pombe*, which is a putative beta subunit of the branched chain alpha-keto acid dehydrogenase E1-beta subunit, caused a septum restoration in the *parA-*overexpressing background mutant. In *S. pombe*, it has been identified that the *pdb1* deletion mutant was lethal after spore germination without cell division and with elongated germ tubes (Cavan and MacDonald, 1994; Beltrao et al., 2009; Kettenbach et al., 2015). Additionally, a previous study has identified that *pdb1* and *par1* are both detected in purified Paa1p-TAP complex by using LC-MS/MS while the A-subunit of the PP2A holoenzyme, Paa1p which has a function of controlling the asymmetric protein localization to old mitotic SPB, is included in SIN-inhibitory phosphatase complex(Singh et al., 2011; Johnson et al., 2012). These data suggest that *pdb1* and *par1* in *S. pombe* might be coordinately regulate the SIN-inhibitory phosphatase complex. Most notably, our data indicate that the *pdbA* deletion in the background of *parA-*overexpressing strain displayed a phenotype of septum restoration under the liquid cultural condition. This finding suggests that the *pdbA* might be also involved in the function of co-regulating cytokinesis with *parA* in the filamentous fungus *A. nidulans*, which has not previously been reported. The possible explanation for this phenotypic phenomenon is that ParA and PdbA might work in a concerted fashion to keeping the SIN proteins on the old SPB. Overexpressing of *parA* may break this asymmetric localization pattern in the old mitotic SPB. In contrast, deletion *pdbA* could rescue the asymmetric protein re-localization on the old SPB resulted from overexpressed *parA*, suggesting *pdbA* has a parallel complementary function with *parA* during septation.

Most interestingly, we found that overexpression of *mztA* could remarkably restore the defective phenotype of the *parA*-overexpressing strain to the wild-type-like septation and conidiation. Moreover, there is evidence that defects induced by overexpressed *parA* are rescued by overexpressed *mztA* in a dose-dependent manner, and deletion of *mztA* restores the abnormal multiple septa of the stalk in the deletion of *parA* to the wild-type non-septum phenotype, which further demonstrates that *mztA* works as a suppressor of *parA* during septation and conidiation in *A. nidulans*.

Additionally, the *mztA* single deletion strain showed a significantly reduced septa phenotype, implying that *mztA* itself may act as a positive regulator for septation in *A. nidulans*. Previous studies in *S. pombe, Drosophila melanogaster, Xenopus laevis*, and *Homo sapiens* have demonstrated that MztA homologous protein could function as a component of the γ-TuRCs (γ-tubulin ring complexes), which is essential for its recruitment to the microtubule-organizing centers (Raff et al., 1993; Stearns and Kirschner, 1994; Hutchins et al., 2010; Dhani et al., 2013). In contrast, γ-TuRC, which consists of γ-tubulin and γ-tubulin complex proteins 2–6 (GCP2–6), possesses a potent microtubule nucleation activity (Stearns and Kirschner, 1994; Gard et al., 1995; Zheng et al., 1995; Masuda et al., 2013). Meanwhile, SPB can act as an MTOC to initiate microtubule nucleation, where the microtubule serves as a track for SIN proteins transporting from SPB to the septum site. The process of γ-TuRCs anchors to SPB and the transportation of the SIN signal pathway to SPB are both required for the septation in *A. nidulans* (Zekert et al., 2010; Johnson et al., 2012; Zhang et al., 2017). Therefore, the abovementioned information suggests that the SPB-localized MztA may also affect cytokinesis/septation through regulating the microtubule nucleation activity. Through Northern blotting we found *parA* was abundantly expressed in all indicated stages, but *mztA* was strongly expressed in G7 and V12, and then weakly expressed in V18 and almost vanished in S24, suggesting that the expression pattern for *mztA* differs substantially from that of *parA* (Figure 4C). Moreover, based on data from Figure 5, deletion of *mztA* showed an impact on conidiophore morphology and colony growth. Therefore, we hypothesize that MztA may regulate the upstream genes of sporulation pathway. Previous studies have identified that not all genes such as *fphA* (phytochrome), *lreA* and *lreB* (blue-light sensor)*, laeA* (methyltransferase) that affect the production of spores will be highly expressed during the process of conidiation (Bok and Keller, 2004; Purschwitz et al., 2008; Etxebeste et al., 2010).

On the other hand, microtubules require polymerization and depolymerization to ensure the balance of the diverse cellular structures and processes in eukaryotes. Previous studies have demonstrated that PP2A, a serine–threonine protein phosphatase, also associates with microtubules. For example, in *Caenorhabditis elegans* embryos, PP2A and its regulatory subunit SUR-6, together with the cortically directed microtubule pulling force, actively disassemble PCM (pericentriolar material) during mitotic exit (Enos et al., 2018). Likewise, in COS cells, okadaic acid, an inhibitor of protein PP2A, could impair the binding ability of tubulin carboxypeptidase to microtubules thereby affecting the stability of MTs (Hiraga and Tamura, 2000; Contín et al., 2003; Nunbhakdi-Craig et al., 2007; Yoon et al., 2008). Thus, based on recent literature and our findings, we conclude that ParA and MztA may cooperate to control the stability of microtubules. MztA may strengthen the stability of MTs to ensure the exact localization of SIN members to SPB, whereas ParA may be able to depolymerize MTs through increasing tubulin carboxypeptidase activity (Hiraga and Tamura, 2000; Tar et al., 2006). To clarify this hypothesis, we further observed the localization of GFP-MobA, a core member of SIN, which has been demonstrated to appear at the spindle pole body (SPB) and the septum site in previous studies. Clearly, the overexpression of *parA* truly causes the abnormal non-SPB localization of GFP-MobA with a cytoplasmic distribution throughout the hyphal cells. In comparison, the double overexpression of *parA* and *mztA* strain showed a wild-type GFP-MobA signal at the SPB and septation site, indicating that the abnormal localization of GFP-MobA induced by over-activated PP2A-ParA, can be rescued by over-expressed MztA. This phenomenon is coincided with the phenotypic rescue experiment observed in the double over-expressed *parA* and *mztA* strain. These findings imply that regulators, which may affect the stability of microtubules, could change the correct localization of the SIN components in the SPB. Consequently, overexpressing *mztA* can suppress the defected septation phenotype induced by overexpressing *parA* probably through maintaining the microtubule balance of polymerization and depolymerization at SPB/MTOC.

## Author contributions

PJ and LL: conception and design of the investigation. PJ and SZ: completion of the experiments. PJ and LL: evaluation and analysis of the results and manuscript writing. PJ, SZ, and LL: final approval of the manuscript.

### Conflict of interest statement

The authors declare that the research was conducted in the absence of any commercial or financial relationships that could be construed as a potential conflict of interest.
