# Supplementary material for: Mitotic-Spindle Organizing Protein MztA Mediates Septation Signaling by Suppressing the Regulatory Subunit of Protein Phosphatase 2A-ParA in Aspergillus nidulans
Source: Front Microbiol. 2018 May 8;9:988. doi: 10.3389/fmicb.2018.00988 (PMC5951981; doi:10.3389/fmicb.2018.00988)
Supplement: Supplementary file 1 [file Data_Sheet_1.PDF]

Figures and legends

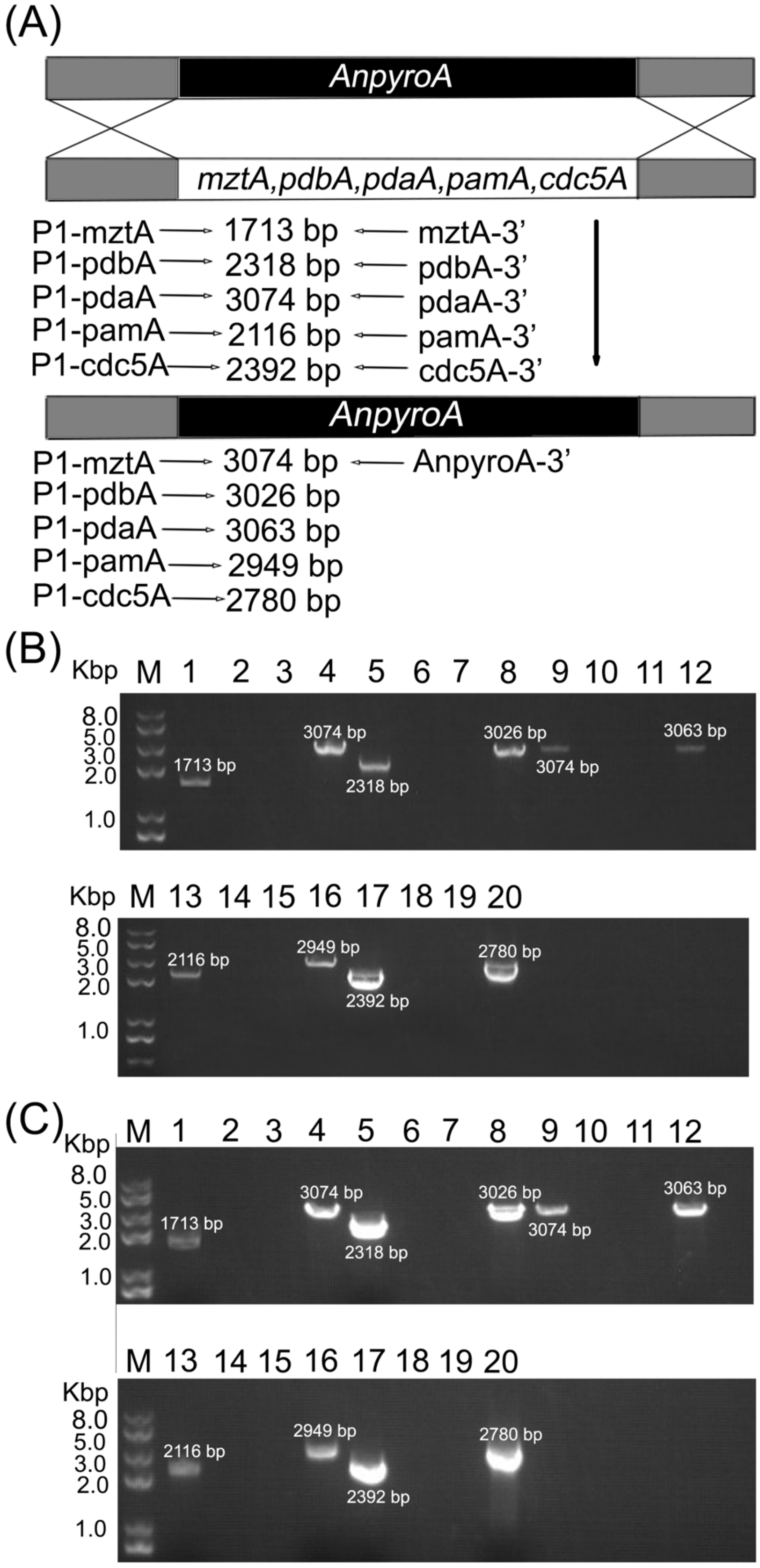

Fig. S1 Schematic diagrams of diagnostic PCR for deletion strains.

(A) Diagrams showing the deletion strategy for the *mztA, pdbA, pdaA, pamA, B* deletion mutants with *AnpyroA* marker in the background of the *parA*-overexpressed strain and the parental wild type (TN02A7).

(B) PCR analysis demonstrated that the *mztA, pdbA, pdaA, pamA, cdc5A* could not be detected, indicating that the integration of the *AnpyroA* nutritional marker into the genome at the original *mztA, pdbA, pdaA, pamA, cdc5A* locus in the *parA*-overexpressed strain. For lanes 1 and 3, the primers were P1-mztA/mztA-3' to detect whether *mztA* still existed in the genome and the expected size was 1713 bp. For lanes 2 and 4, the primers were P1-mztA/AnpyroA-3' to detect whether there was a homologous recombination to replace *mztA* with the nutritional marker gene *AnpyroA* in the genome, and the expected size was 3074 bp. For lanes 5 and 7, the primers were P1- pdbA/pdbA-3' to detect whether *pdbA* still existed in the genome and the expected size was 2318 bp. For lanes 6 and 8, the primers were P1-pdbA/AnpyroA-3' to detect whether there was a homologous recombination to replace *pdbA* with the nutritional marker gene *AnpyroA* in the genome, and the expected size was 3026 bp. For lanes 9 and 11, the primers were P1- pdaA/pdaA-3' to detect whether *pdaA* still existed in the genome and the expected size was 3074 bp. For lanes 10 and 12, the primers were P1-pdaA/AnpyroA-3' to detect whether there was a homologous recombination to replace *pdaA* with the nutritional marker gene *AnpyroA* in the genome, and the expected size was 3063 bp. For lanes 13 and 15, the primers were P1- pamA/pamA-3' to detect whether *pamA* still existed in the genome and the expected size was 2116 bp. For lanes 14 and 16, the primers were P1-pamA/AnpyroA-3' to detect whether there was a homologous recombination to replace *pamA* with the nutritional marker gene *AnpyroA* in the genome, and the expected size was 2949 bp. For lanes 17 and 19, the primers were P1-cdc5A/cdc5A-3' to detect whether *cdc5A* still existed in the genome and the expected size was 2392 bp. For lanes 18 and 20, the primers were P1- cdc5A/AnpyroA-3' to detect whether there was a homologous recombination to replace *cdc5A* with the nutritional marker gene *AnpyroA* in the genome, and the expected size was 2780 bp. In lanes 1, 2, 5, 6, 9, 10, 13, 14, 17, and 18, the genomic DNA of the *parA*-overexpressed mutant was used for the PCR templates; lanes 3 and 4 used the genomic DNA of transformants of OE:: $parA^{\Delta mztA}$ as PCR templates; lanes 7 and 8 used the genomic DNA of transformants of OE:: $parA^{\Delta pdbA}$ as PCR templates; lanes 11 and 12 used the genomic DNA of transformants of OE:: $parA^{\Delta pdaA}$ as PCR templates; lanes 15 and 16 used the genomic DNA of transformants of OE:: $parA^{\Delta pamA}$ as PCR templates; lanes 19 and 20 used the genomic DNA of transformants of OE:: $parA^{\Delta cdc5A}$ as PCR templates.

(C) PCR analysis demonstrated that the *mztA, pdbA, pdaA, pamA,* and *cdc5A* could not be detected, indicating that the integration of the *AnpyroA* nutritional marker into the genome at the original *mztA, pdbA, pdaA, pamA,* and *cdc5A* locus in the parental wild type strain. For lane 1 and 3, the primers were P1-mztA/mztA-3' to detect whether *mztA* still existed in the genome and the expected size was 1713 bp. For lanes 2 and 4, the primers were P1-mztA/AnpyroA-3' to detect whether there was a homologous recombination to replace *mztA* with the nutritional marker gene *AnpyroA*

in the genome, and the expected size was 3074 bp. For lanes 5 and 7, the primers were P1- pdbA/pdbA-3' to detect whether *pdbA* still existed in the genome and the expected size was 2318 bp. For lanes 6 and 8, the primers were P1-pdbA/AnpyroA-3' to detect whether there was a homologous recombination to replace *pdbA* with the nutritional marker gene *AnpyroA* in the genome, and the expected size was 3026 bp. For lanes 9 and 11, the primers were P1- pdaA/pdaA-3' to detect whether *pdaA* still existed in the genome and the expected size was 3074 bp. For lanes 10 and 12, the primers were P1-pdaA/AnpyroA-3' to detect whether there was a homologous recombination to replace *pdaA* with the nutritional marker gene *AnpyroA* in the genome, and the expected size was 3063 bp. For lanes 13 and 15, the primers were P1- pamA/pamA-3' to detect whether *pamA* still existed in the genome and the expected size was 2116 bp. For lanes 14 and 16, the primers were P1-pamA/AnpyroA-3' to detect whether there was a homologous recombination to replace *pamA* with the nutritional marker gene *AnpyroA* in the genome, and the expected size was 2949 bp. For lanes 17 and 19, the primers were P1-cdc5A/cdc5A-3' to detect whether *cdc5A* still existed in the genome and the expected size was 2392 bp. For lanes 18 and 20, the primers were P1- cdc5A/AnpyroA-3' to detect whether there was a homologous recombination to replace *cdc5A* with the nutritional marker gene *AnpyroA* in the genome, and the expected size was 2780 bp. In the lanes 1, 2, 5, 6, 9, 10, 13, 14, 17, and 18, the genomic DNA of the parental wild type (TN02A7) was used for the PCR template; lanes 3 and 4 used the genomic DNA of transformants of *ΔmztA* as PCR templates; lanes 7 and 8 used the genomic DNA of transformants of *ΔpdbA* as the PCR templates; lanes 11 and 12 used the genomic DNA of the transformants of *ΔpdaA* as PCR templates; lanes 15 and 16 used the genomic DNA of transformants of *ΔpamA* as PCR templates; lanes 19 and 20 used the genomic DNA of transformants of *Δcdc5A* as PCR templates.

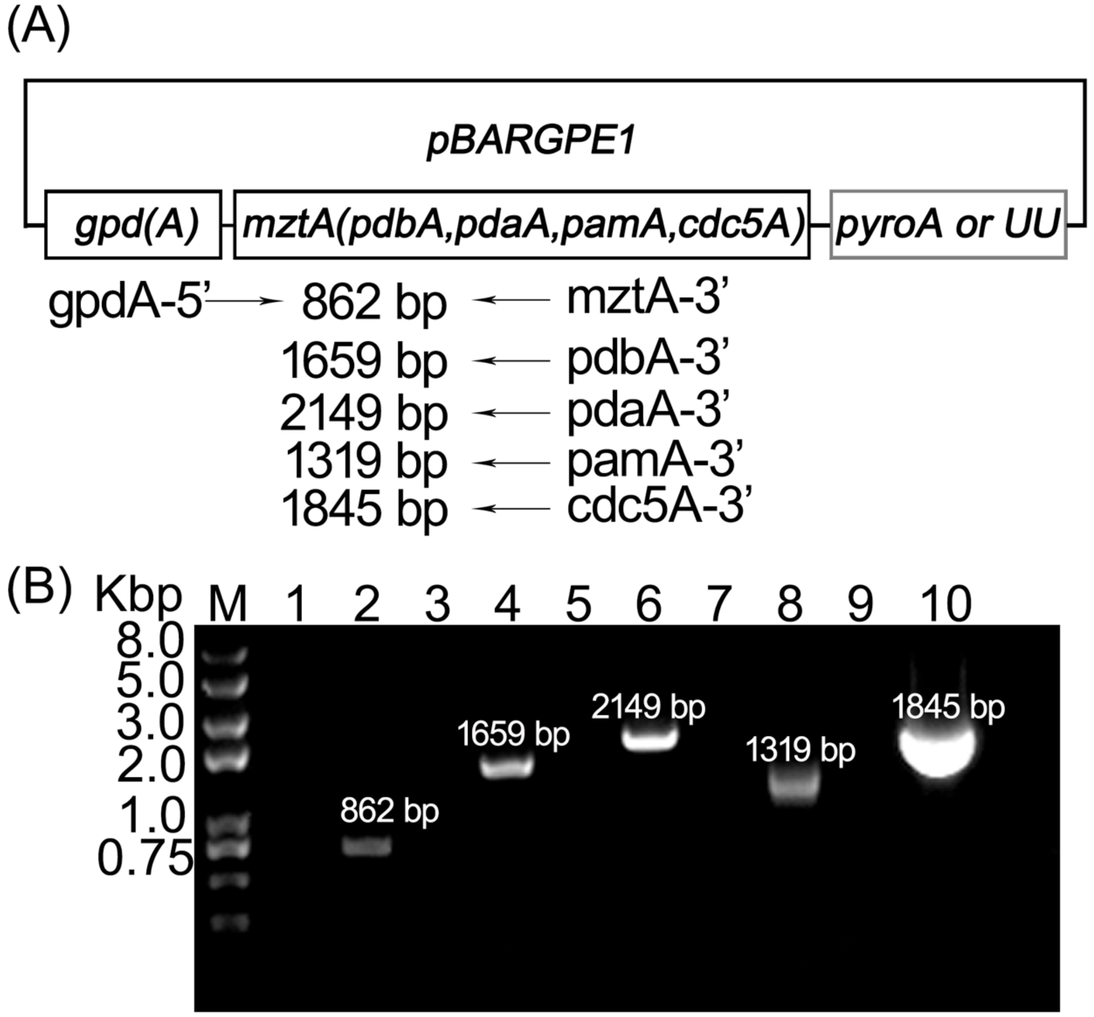

Fig. S2 Schematic diagrams of the diagnostic PCR for the overexpression strains.
(A) Diagrams showing the strategy for generating and verifying the overexpression of *mztA, pdbA, pdaA, pamA,* and *cdc5A* mutants in the background of *parA*-overexpressed strains.
(B) PCR analysis showed that the overexpression gene fragment of *mztA, pdbA, pdaA, pamA,* and *cdc5A* were detected in the *parA*-overexpressed strain. For lane 1 and 2, the primers were gpdA-5'/mztA-3' to detect whether *gpdA-mztA* existed in the genome and the expected size was 862 bp; for lanes 3 and 4, the primers were gpdA-5'/pdbA-3' to detect whether *gpdA-pdbA* existed in the genome and the expected size was 1659 bp; for lanes 5 and 6, the primers were gpdA-5'/pdaA-3' to detect whether *gpdA-pdaA* existed in the genome and the expected size was 2149 bp; for lanes 7 and 8, the primers were gpdA-5'/pamA-3' to detect whether *gpdA-pamA* existed in the genome and the expected size was 1319 bp; for lanes 9 and 10, the primers were gpdA-5'/cdc5A-3' to detect whether *gpdA-cdc5A* existed in the genome and the expected size was 1845 bp; In lanes 1, 3, 5, 7, and 9, the genomic DNA of the *parA*-overexpressed strain was used for the PCR template; for lanes 2, 4, 6, 8, and 10 the genomic DNA of transformants of OE::$parA^{OE::mztA}$, OE::$parA^{OE::pdbA}$, OE::$parA^{OE::pdaA}$, OE::$parA^{OE::pamA}$, OE::$parA^{OE::cdc5A}$ mutants was used as the PCR

templates, respectively.

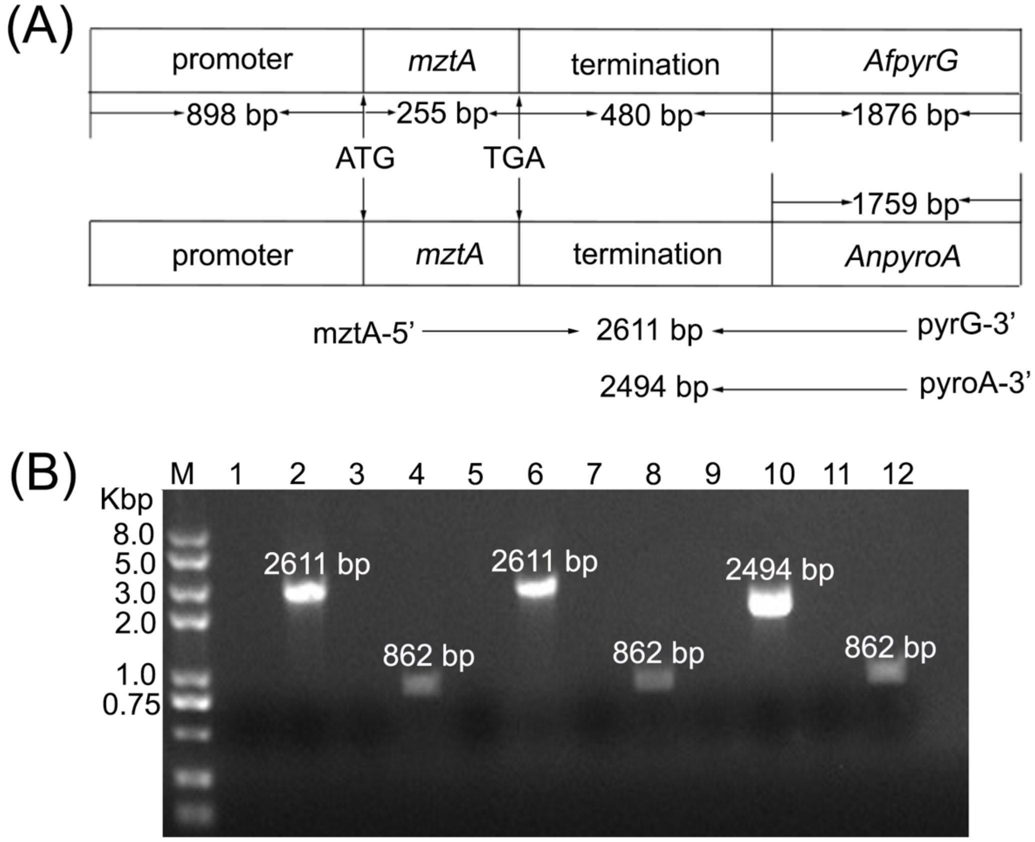

Fig. S3 Schematic diagrams of the diagnostic PCR for the complementation strains.
(A) Illustration of the constitutive parental wild type (TN02A7) *mztA* gene driven by the endogenous promoter with two different nutritional markers, *AfpyrG* and *AnpyroA*.
(B) PCR analysis showed that the overexpression of *mztA* by the endogenous promoter and the constitutive promoter (*gpdA*) was detected in *ΔmztA*, OE::*parA* and the parental wild-type strains,. For lanes 1, 2, 5, and 6, the primers were mztA-5'/ pyrG-3' to detect whether *mztA* controlled by the endogenous promoter with *AfpyrG* marker existed in the genome of the parental wild-type (TN02A7) and *ΔmztA* mutant, and the expected size was 2611 bp; for lanes 9 and 10, the primers were mztA-5'/ pyroA-3' to detect whether *mztA* controlled by the endogenous promoter with *AnpyroA* marker existed in the genome and the OE::*parA* strain, and the expected size was 2494 bp; for lanes 3, 4, 7, 8, 11, and 12, the primers were gpdA-5'/mztA-3' to detect whether *gpdA*-*mztA* existed in the genome and the expected size was 862 bp. For lanes 1 and 3, the genomic DNA of the parental wild type (TN02A7) was used as the PCR template; lane 2 used the genomic DNA of transformant of WT^{mztA}; lane 4 used the genomic DNA of transformant of $\mathrm{WT}^{\mathrm{OE::}mztA}$; for lanes 5 and 7, the genomic DNA of the *ΔmztA* strain was used for the PCR template; lane 6 used the genomic DNA of transformant of $\Delta mztA^{mztA}$ mutant; lane 8 used the genomic DNA of

transformant of *ΔmztA* $^{OE::mztA}$ mutant; for lanes 9 and 11, the genomic DNA of the OE::*parA* strain was used for the PCR template; lane 10 used the genomic DNA of the transformant of OE::*parA*^{mztA} mutant; lane 12 used the genomic DNA of the transformant of OE::*parA*$^{OE::mztA}$ mutant.

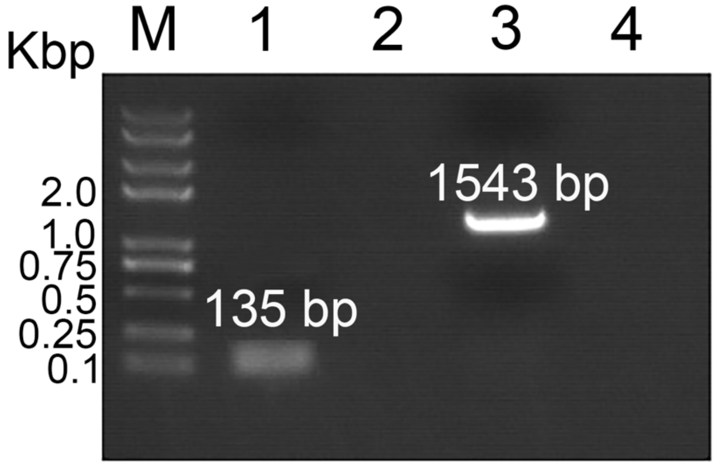

Fig. S4 PCR analysis demonstrated that *parA* and *mztA* could not detected in the *ΔparAΔmztA* double-deletion mutant. For lanes 1 and 2, the primers were Diag-mztA-5'/ Diag-mztA-3' to detect whether *mztA* existed in the genomic DNA and the expected size was 135 bp. For lanes 3 and 4, the primers were Diag-parA-5'/ Diag-parA-3' to detect whether *parA* existed in the genomic DNA and the expected size was 1543 bp. For lanes 1 and 3, the genomic DNA of the parental wild type (TN02A7) was used for the PCR template; For lanes 2 and 4, the genomic DNA of the *ΔparAΔmztA* double-deletion mutant was used for the PCR template.

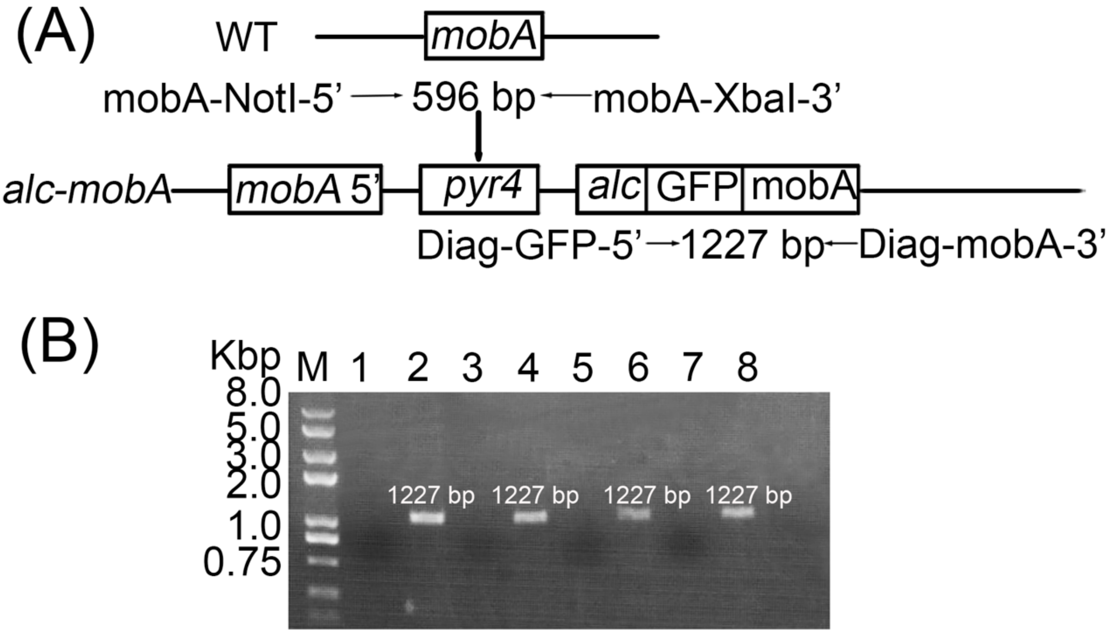

Fig. S5 Schematic diagrams of diagnostic PCR for conditional *alc(p)-GFP-mobA* strain in the background of the parental wild type (R21), *mztA*-overexpressed mutant, *parA*-overexpressed mutant and the double overexpression of *parA* and *mztA* mutant.

(A) Diagram showing the strategy for generating and verifying *alc(p)-GFP-mobA* strain by diagnostic PCR.

(B) PCR analysis displayed that in the parental wild type (R21), *mztA*-overexpressed mutant, *parA*-overexpressed mutant and the double overexpression of *parA* and *mztA* mutant exist the *alc(p)-GFP-mobA* fragment. For lanes 1, 2, 3, 4, 5, 6, 7, and 8, the primers were Diag-GFP-5'/ Diag-mobA-3' to detected the *alc(p)-GFP-mobA* fragment exist in the genomic DNA, and the expected size was 1227 bp. For the lanes 1 and 2, the genomic DNA of the parental wild type(R21) was used for PCR template; for the lanes 3 and 4, the genomic DNA of the *mztA*-overexpressed mutant was used for PCR template; for the lanes 5 and 6, the genomic DNA of the *parA*-overexpressed mutant was used for PCR template; for the lanes 7 and 8, the genomic DNA of the double overexpression *parA* and *mztA* mutant was used for PCR template.
